# Robotic Intracorporeal Single-Stapled Anastomosis (RISS) is Associated with Lower Anastomotic Leakage Rates than the Double-Stapled Technique After Minimally Invasive Total Mesorectal Excision for Rectal Cancer

**DOI:** 10.1245/s10434-025-18742-3

**Published:** 2025-11-20

**Authors:** Paulo Roberto Stevanato Filho, Tiago Santoro Bezerra, Tomas Mansur Duarte Miranda Marques, Renata Mayumi Takahashi, Rebeca Hara Nahime, Bruna Elisa Catin Kupper, Wilson Toshihiko Nakagawa, Ademar Lopes, Samuel Aguiar Junior

**Affiliations:** Colorectal Cancer Reference Center, A.C. Camargo Cancer Center, R. Professor Antônio Prudente, São Paulo, SP Brazil

**Keywords:** Rectal cancer, Total mesorectal excision, Anastomotic leak, Robotic surgery, Single-stapled anastomosis

## Abstract

**Background:**

Alternatives to the double-stapled (DS) technique for creating anastomoses after minimally invasive total mesorectal excision (TME) have been proposed to reduce complications and costs. Robotic intracorporeal single-stapled anastomosis (RISS) was developed as a technically intuitive approach. Standardizing such an intracorporeal robotic technique—which achieves adequate pelvic exposure, precise rectal transection, and secure anastomosis construction—may optimize outcomes, particularly anastomotic leakage (AL).

**Methods:**

A cohort study was conducted using our prospective institutional database and included patients < 80 years who underwent minimally invasive elective TME for extraperitoneal rectal cancer. Patients were allocated to the DS (abdominal stapled transection with double-stapled anastomosis) or RISS (robotic intracorporeal rectal transection with single-stapled anastomosis) groups. The exclusion criteria were nonrestorative procedures, intersphincteric resection, open surgery, and no indocyanine green perfusion assessments. The primary endpoint was 90-day clinical or radiological AL.

**Results:**

Among 380 TMEs, 167 met the inclusion criteria (71 RISS; 96 DS). The 90-day AL rate was significantly lower in the RISS group (5.6% vs. 16.7%; *p *= 0.032). Reintervention (1.4% vs. 10.4%; *p *= 0.025), overall morbidity (33.3% vs. 52.5%; *p *= 0.014), and length of stay (*p *< 0.0001) were lower following RISS. Multivariable analysis revealed that DS technique (odds ratio [OR] 3.3; *p *= 0.038) and comorbidities (OR 3.1; *p *= 0.028) independently predicted AL. Each additional stapler firing increased the risk of AL (OR 1.62; *p *= 0.016), and ≥3 firings predicted AL (OR 4.92; *p *= 0.011).

**Conclusions:**

Compared with DS, RISS was associated with lower anastomotic leakage, morbidity, and reintervention and shorter hospitalization. This standardized robotic approach is safe, reproducible, and potentially cost effective.

**Supplementary Information:**

The online version contains supplementary material available at 10.1245/s10434-025-18742-3.

Technical alternatives associated with total mesorectal excision (TME) have been developed to reduce anastomotic complications, ensure adequate distal margins, and minimize costs by avoiding the need for linear stapler use. Single-stapled anastomosis (SS) has been identified as a safe alternative to the standard double-stapled (DS) technique.^1–3^ However, standardizing a robotic-assisted intracorporeal technique—which can ensure optimal pelvic exposure, precise rectal transection, and secure anastomosis creation—is crucial for further optimizing short-term oncologic and functional outcomes, including the incidence of anastomotic leakage (AL).

Total mesorectal excision for extraperitoneal rectal cancer often requires low colorectal anastomosis because of the need for complete mesorectal fascia excision, a key factor for achieving adequate locoregional oncologic control.^4–7^ In the DS technique, a linear stapler is applied to the distal rectum “blindly,” often obliquely, owing to the near impossibility of achieving perfect right-angle placement, particularly in patients with a narrow pelvis. Multiple stapler firings are frequently needed, a factor that has been identified as an independent risk factor for AL and other adverse outcomes.^8–10^

Alternative techniques, such as TaTME or transanal transection with single-stapled anastomosis (TTSS), allow direct visual control of the rectal transection process and have produced adequate distal margins without the need for double stapling.^2^^,^^3^^,^^11–13^

Recently, prospective studies and randomized trials have demonstrated superior local oncologic control with robotic-assisted surgery.^14^ Within this context, integrating the concept of single-stapled anastomosis into the robotic environment is a natural technical evolution. Robotic intracorporeal single-stapled anastomosis (RISS) was standardized in our institution through a stepwise protocol, achieving optimal endopelvic exposure and safe tumor-free rectal transection under direct vision, followed by the creation of an anastomosis with a hand-sewn purse-string. By standardizing the technique, a uniform, well-perfused anastomotic ring can be created without irregularities associated with cross-stapling. Since its implementation, RISS has become the preferred reconstructive strategy and is routinely adopted whenever a restorative procedure following TME is planned.^15^

## Methods

### Study Design

Data from patients who underwent minimally invasive total mesorectal excision (MI-TME) for extraperitoneal rectal cancer were analyzed. The prospective cohort included patients who underwent RISS from December 2022 to April 2025, while the control group consisted of patients who underwent double-stapled (DS) anastomosis since January 2018. All data were extracted from our prospective institutional database.

All procedures were performed by the same surgical team, composed of surgeons with more than five years of experience in minimally invasive rectal surgery, with at least 30 procedures (up to 200), both robotic and laparoscopic. The RISS technique was introduced in December 2022 as an institutional innovation and alternative to the conventional double-stapling method. During the overlap period (January 2023–May 2024), both techniques were performed concurrently as part of the institutional transition process. From June 2024 onward, RISS became the standard reconstructive approach at our center.

This design allowed both techniques to be analyzed within their respective real-world phases of clinical application, minimizing transitional overlap bias. The choice between techniques was not based on patient characteristics or surgeon preference but reflected the progressive adoption of RISS as surgeons became familiar with the procedure.

In line with the International Consensus on Reporting Anastomotic Leaks After Colorectal Cancer Surgery,^16^ anastomotic leakage classification and reporting were performed following standardized recommendations, ensuring that our findings were comparable and reproducible.

### Eligibility Criteria

Consecutive patients aged ≤ 80 years who underwent elective MI-TME with diverting stoma, all operated on by the same surgical team, were allocated into two groups:

- RISS: Robotic intracorporeal single-stapled anastomosis with intracorporeal rectal transection.
- DS: Linear rectal transection with transabdominal stapling and DS anastomosis.

The exclusion criteria were nonrestorative procedures (abdominoperineal resection (APR) or Hartmann), intersphincteric resection with coloanal anastomosis, open surgery, and the absence of indocyanine green fluorescence in the assessment of distal colon perfusion.^17^^,^^18^

### Steps for Intra-abdominal Rectal Transection

**Fig. 1:**
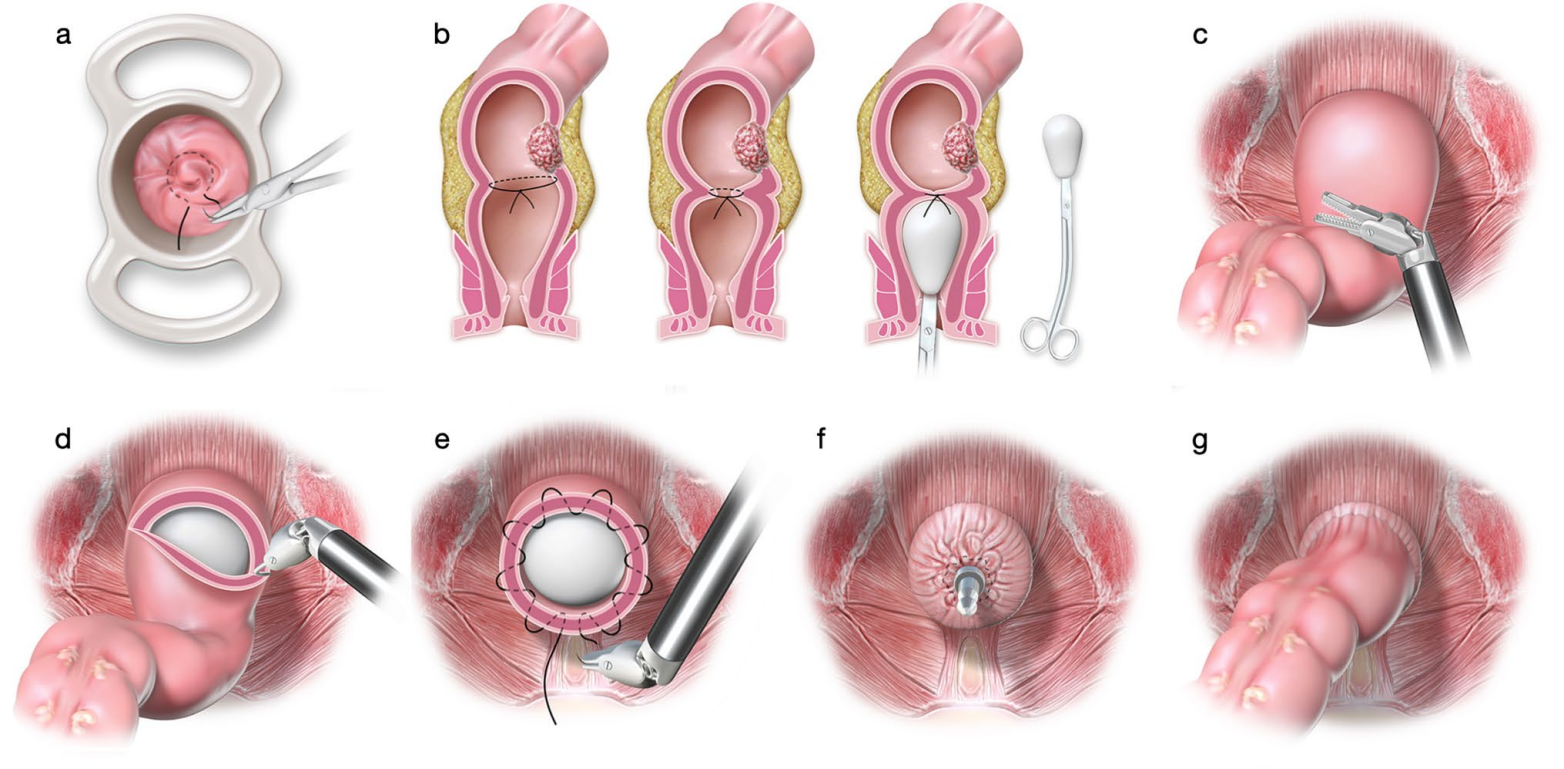
Steps of RISS. **a** A transanal intraluminal purse-string suture was applied before starting the operation with 2.0 suture or similar, with a safety margin leaving the distal rectum completely free of tumor. The distal rectal stump is washed with 0.9% saline solution and chlorhexidine; **b** After total mesorectal dissection and complete left colon mobilization, the rectal transection proceeds. To facilitate perfect exposure and visualization of the closed free rectal stump, the auxiliary surgeon introduces a plug; **c** Intracorporeal exposure area for rectal transection; **d** Precise lower rectal transection and endorectal plug exposure; **e** Robotic intracorporeal purse-string suture of the rectal stump; **f** Circular stapler was inserted into the rectum; **g** Single-stapled anastomosis is performed

**Fig. 2:**
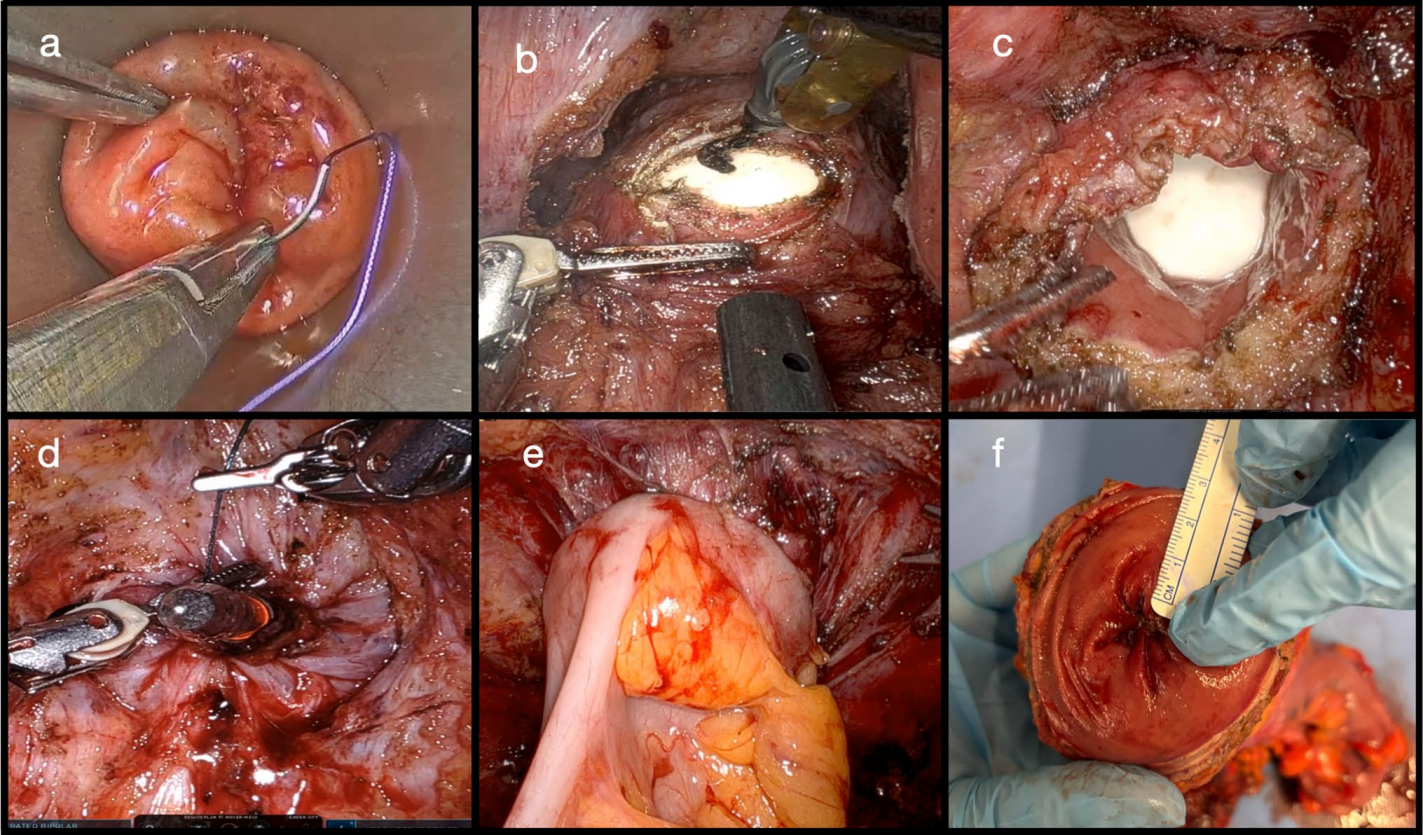
Stepwise sequence for intra-abdominal rectal transection and creation of a hand-sewn purse-string anastomosis; **a** Transanal intraluminal purse-string suture isolating completely the distal rectal stump; **b** Adequate exposure of the rectal transection area; **c** Precise distal rectal transection with endorectal plug exposure; **d** Intracorporeal purse-string suture of the rectal stump followed by introduction of the circular stapler into the rectum; **e** Construction of the single-stapled anastomosis; **f** Circumferential distal rectal margin

Before the abdominal procedure was initiated, a transanal intraluminal purse-string suture was systematically applied immediately distal to the lesion using a 2-0 suture or equivalent material, with the objective of isolating the longest possible segment of the tumor-free distal rectum (Figs. 1, 2; Supplemental Video 1). This step was performed with a permanent cylindrical trunk anoscope (TEO Platform, Karl Storz), allowing rapid execution, typically in less than 10 min. Both the permanent cylindrical trunk anoscope (> 34 mm in diameter) and disposable alternatives, such as the CK34M model (Frankenman Int. Ltd.), are suitable options for transanal purse-string suturing.^15^

Following suture completion, the distal rectal stump was irrigated with 0.9% saline solution combined with chlorhexidine. The patient was then positioned in a modified lithotomy, Trendelenburg, and right-side-down position, with the pneumoperitoneum maintained at 12 mmHg. The robotic platform was subsequently docked. The robotic approach to total mesorectal excision (TME) was conducted according to previously described standardized techniques.

After full mobilization of the left colon and complete mesorectal dissection, rectal transection was performed. To optimize visualization and provide adequate exposure of the closed distal rectal stump, an auxiliary surgeon introduced a handmade plug (composed of gauze wrapped in a sterile surgical glove; Figs. 1, 2; Supplemental Video 1). The entire distal rectal segment was presented transanally to facilitate precise low transection under direct endopelvic visualization. In accordance with oncological principles for rectal cancer surgery, a distal resection margin of > 2 cm was prioritized. For distal rectal tumors, especially after neoadjuvant treatment, efforts were made to achieve a distal margin of > 1.0 cm to allow the subsequent creation of a circular stapled anastomosis.

The same monopolar hook cautery and robotic scissors used during mesorectal dissection were employed for rectal transection, without the need for additional instruments. The robotic platform facilitated precise identification and dissection of a safe transection plane, defined by a macroscopically clear and homogeneous margin, which was immediately irrigated following completion of the initial intraluminal purse-string suture.^15^

### Steps for Creating an Intracorporeal Hand-Sewn Purse-string Anastomosis

In all procedures, perfusion of the distal colon was systematically assessed using indocyanine green fluorescence angiography. After adequate perfusion was confirmed, a purse-string suture was placed on the exteriorized proximal colon for positioning of the anvil of a 29/31 mm circular stapler, followed by reintroduction of the segment into the pelvic cavity.

Intracorporeally, via robotic pelvic access, purse-string invagination was performed in the rectal stump to prepare for the introduction of the circular stapler (Fig. 2). Following its introduction, the stapler trocar was opened, and the previously constructed purse-string suture was meticulously secured over the trocar. To facilitate progressive and uniform tightening of the purse-string, unidirectional barbed sutures (2-0, 26 mm needle, 15 cm in length) were preferred (Figs. 1, 2; Supplemental Video 1).^15^

### Endpoints

The primary endpoint was the occurrence of clinical or radiological anastomotic leakage (AL) within 90 days postoperatively. Anastomotic leakages were classified as early (≤30 days) and graded according to the International Study Group of Rectal Cancer (ISREC) and the severity system proposed by the International CoReAL Consensus.^10^^,^^16^ Postoperative complications were reported according to the Clavien–Dindo classification.^19^

Preoperative tumor staging was performed in accordance with the TNM guidelines. Indications for neoadjuvant chemoradiotherapy and total neoadjuvant therapy were discussed systematically at multidisciplinary tumor board meetings for all patients with locally advanced rectal cancer (stage II: T3/4N0M0; stage III: any T, N+, M0), as assessed by contrast-enhanced pelvic magnetic resonance imaging in accordance with National Comprehensive Cancer Network recommendations.^20^ Tumor location was defined according to current guidelines: low rectum for tumors fully or partially below the origin of the levator ani, up to 5 cm from the anal verge; mid-rectum for tumors > 5 cm from the anal verge.^20^

After the completion of neoadjuvant therapy, restaging was performed, and surgery was scheduled. Pathological staging followed the AJCC Cancer Staging System, Version 9 for Rectal Cancer.^21^ Clinical-oncological follow-up adhered to institutional protocols, with regular consultations and examinations according to patient-specific needs.

### Statistical Analysis

Categorical and dichotomous variables are expressed as frequencies and percentages. Continuous variables were assessed for normality using the Shapiro‒Wilk test (in which *P *< 0.05 indicated a nonnormal distribution) and are presented as the mean ± standard deviation or median with interquartile range (IQR), as appropriate. Group comparisons were conducted using Pearson’s chi-square or Fisher’s exact tests for categorical variables and unpaired *t*-tests or Mann–Whitney *U* tests for continuous variables, according to the data distribution.

To identify independent factors associated with anastomotic leakage, a multivariable logistic regression model was constructed, including variables significant in univariable analysis (*P *< 0.05) and established risk factors reported in the literature.^22^ The results are presented as odds ratios (ORs) with 95% confidence intervals (CIs). All tests were two-sided, and statistical analyses were performed using IBM SPSS Statistics, version 31 (IBM Corp., Armonk, NY).

To assess the learning curve of the RISS technique, a cumulative sum (CUSUM) analysis of operative time was performed. Operative times were plotted sequentially according to case order, and the cumulative deviations from the mean operative time were calculated and displayed as a CUSUM chart (Supplementary Fig. 1).

## Results

### Patient Characteristics

Among 380 TMEs performed during the study period, 167 patients met the inclusion criteria (71 RISS and 96 DS). Baseline characteristics—including sex, age, body mass index, smoking status, American Society of Anesthesiologists (ASA) physical status, and relevant comorbidities—were comparable between the groups (Table 1).^23^

**Table 1:** Demographics and baseline characteristics

Data	RISS, n (%)	DS, n (%)	*P*
No. patients	71	96	
Sex, females	20 (28.2)	35 (36.5)	0.318
Age, years, mean ± SD	55.6 ± 11.4	58.3 ± 12.1	0.145
BMI, kg/m^2^, mean ± SD	27.9 ± 4.2	25.1 ± 4.1	0.004
Smoking			
Ex-smokers	6 (8.5)	6 (6.3)	0.763
Active smokers	6 (8.5)	7 (7.3)	0.779
ASA classification			0.088
ASA I	7 (9.9)	17 (17.7)	
ASA II	50 (70.4)	70 (72.9)	
ASA III	14 (19.7)	8 (8.3)	
ASA IV	0	1 (1)	
Relevant comorbidities	32 (45.1)	45 (46.9)	0.876
Diabetes	12 (16.9)	20 (20.8)	0.557
Hypertension	27 (38)	33 (34.4)	0.629
Cardiovascular diseases	4 (5.6)	6 (6.3)	
Pulmonary diseases	1 (1.4)	3 (3.1)	
Thrombosis	1 (1.4)	1 (1)	
Renal diseases	–	2 (2)	
Gastrointestinal disorders	1 (1.4)	–	
Hematology	3 (4.2)	3 (4.2)	

*ASA* American society of Anesthesiologists; *BMI* body mass index; *DS* double-stapled; *RISS* robotic intracorporeal single-stapled anastomosis

Normally distributed variables were analyzed using the unpaired *t-*test, whereas nonnormally distributed variables were analyzed with the Mann‒Whitney *U* test

Categorical variables were assessed with the chi-square test or Fisher’s exact test

### Tumor Characteristics and Operative Outcomes

The proportion of patients who underwent neoadjuvant therapy, preoperative tumor stage, number of harvested lymph nodes, intraoperative complications, and number of intraoperative blood transfusions were similar between the groups (Table 2). Compared with those of the DS group, the tumors in the RISS group were located closer to the anal verge (5.08 ± 2.32 cm vs. 6.27 ± 2.11 cm; *P *< 0.001), and the operative time was longer (327 ± 59 min vs. 294 ± 62 min; *P *< 0.001). Protective ileostomy was systematically performed in both groups.

**Table 2:** Tumor characteristics and operative data

Characteristic	RISS, n (%)	DS, n (%)	*P*
Number of patients	71	96	
Tumor distance from the dentate line, cm, mean ± SD	5.08 ± 2.32	6.27 ± 2.11	<0.001
Middle rectum > 5.0 cm	29 (40.8)	58 (60.4)	0.018
Distal rectum ≤ 5.0 cm	42 (59.2)	38 (39.6)	0.018
No. stapler firings for rectal transection, mean ± SD	–	2.42 ± 0.65	
Neoadjuvant therapy	38 (53.5)	65 (67.7)	0.077
Neoadjuvant radiotherapy	35 (49.3)	62 (64.6)	0.057
Clinical tumoral stage			0.216
Stage I	23 (32.4)	19 (19.8)	
Stage II	6 (8.5)	12 (12.5)	
Stage III	41 (57.7)	61 (63.5)	
Stage IV	1 (1.4)	4 (4.2)	
No. lymph nodes	20.09	21.94	0.313
Video assisted	–	43 (44.8)	<0.001
Robot assisted	71	53 (55.2)	
Operative time, min, mean ± SD	327 ± 59	294 ± 62	<0.001
Intraoperative complications	1 (1.4)	2 (2.1)	1.000

*DS* double-stapled; *RISS* robotic intracorporeal single-stapled anastomosis; *SD* standard deviation

Normally distributed continuous variables were analyzed using the unpaired two-sided *t*-test, whereas nonnormally distributed continuous variables were analyzed with the unpaired, two-sided Mann‒Whitney *U* test

Categorical and dichotomous variables were analyzed using the chi-square test or Fisher’s exact test, as appropriate

A CUSUM analysis of operative time demonstrated an initial upward phase, progressive improvement, and a plateau consistent with procedural proficiency (Supplementary Fig. 1).

### Postoperative Outcomes

The 90-day AL rate was significantly lower in the RISS group than in the DS group (5.6% vs. 16.7%; *P *= 0.032). Leakages were stratified by grade (A, B, C) and by timing (early  ≤ 30 days, late > 30 days).

Reinterventions were more frequent in the DS group (10.4 vs. 1.4%; *P *= 0.025). Overall postoperative complications, according to the Clavien–Dindo classification, were more common in the DS group (52.5% vs. 33.3%; *P *= 0.014), while paralytic ileus was the most common complication in both cohorts. Although grade III–IV complications were more common in the DS group, the difference did not reach statistical significance.

The median postoperative length of stay was significantly shorter in the RISS group (*P *< 0.0001). No differences were observed regarding hospital readmission, postoperative blood transfusion, or timely stoma closure (Table 3).

**Table 3:** Postoperative outcomes

Outcomes	RISS, n (%)	DS, n (%)	*P*
No. patients	71	96	
Length of postoperative stay, d, median (IQR)	6 (3–21)	8 (3–43)	0.041
90-day anastomotic leakages	4 (5.6)	16 (16.7)	0.032
Anastomotic leakage timing			0.079
Early (< 30 days)	4 (5.6)	14 (14.6)	
Late (> 30 days)	–	2 (2.1)	
Anastomotic leakage grade			0.121
A	2 (2.8)	6 (6.3)	
B	2 (2.8)	5 (5.2)	
C	–	5 (5.2)	
Reintervention	1 (1.4)	10 (10.4)	0.025
90-day complications	24 (33.3)	53 (52.5)	0.014
90-day postoperative complications			0.159
I	3 (4.2)	4 (4.2)	
II	16 (22.5)	29 (30.2)	
IIIa	2 (2.8)	8 (8.3)	
IIIb	5 (7)	6 (6.3)	
IV	–	3 (3.1)	
Clavien–Dindo Grade III–IV	5 (7)	18 (17.7)	0.063
Postoperative transfusions	2 (2.8)	1 (1)	0.575
Stoma closed	70 (98.6)	94 (97.9)	0.612
Readmission	4 (5.6)	12 (12.5)	0.185

*DS* double-stapled; *RISS* robotic intracorporeal single-stapled anastomosis

Continuous normally distributed variables were analyzed with the unpaired two-sided *t*-test; continuous nonnormally distributed variables were analyzed with the unpaired two-sided Mann‒Whitney *U* test

Categorical and dichotomous variables were analyzed with the chi-square test or Fisher’s exact test

### Pathology Data

The pathological tumor stage was comparable between the groups. No positive distal margins were reported in any patient, and the rate of radial margin positivity did not differ (Table 4).

**Table 4:** Pathology data

Outcomes	RISS, n (%)	DS, n (%)	*P*
Patients, (n)	71	96	
Tumoral stage (T)			0.527
Tis	1 (1.4)	2 (2)	
T0	9 (12.7)	17 (16.8)	
T1	13 (18.2)	10 (9.9)	
T2	16 (22.5)	23 (22.8)	
T3	25 (35.2)	43 (42.6)	
T4	1 (1.4)	0	
Nodes stage (N)			0.210
N0	54 (76.1)	64 (63.4)	
N1	11 (15.5)	24 (23.8)	
N2	6 (8.5)	13 (12.9)	
Metastasis stage (M)			1.000
M0	68 (95.8)	96 (95)	
M1	3 (4.2)	5 (5)	
Positive circumferential margin (tumor deposits)	–	1 (1)	1.000

*DS* double-stapled; *RISS* robotic intracorporeal single-stapled anastomosis

Continuous normally distributed variables were analyzed with the unpaired two-sided *t*-test

Continuous nonnormally distributed variables were analyzed with the unpaired two-sided Mann‒Whitney *U* test

Categorical and dichotomous variables were analyzed with the chi-square test or Fisher’s exact test

### Risk Factors for Anastomotic Leakage

According to the univariable analysis, RISS was protective against AL (OR 0.29; 95% CI 0.09–0.93; *P *= 0.038), whereas clinically relevant comorbidities increased the risk (OR 3.16; 95% CI 1.13–8.54; *P *= 0.002). Multivariable analysis confirmed that the use of RISS (OR 0.30; 95% CI 0.09–0.97; *P *= 0.045) and clinically relevant comorbidities (OR 3.16; 95% CI 1.13–8.81; *P *= 0.028) were independent predictors of AL. Neoadjuvant radiotherapy, which is known to increase the risk of AL in patients with low rectal tumors,^24^^,^^25^ was included in the model, even though it did not reach statistical significance in this cohort (Table 5).

**Table 5:** Univariable and multivariable logistic regression analyses of the risk factors for 90-day AL

Variable	Univariable analysis		Multivariable analysis	
	OR/MD (95% CI)	*P*	OR (95% CI)	*P*
Anastomosis type (vs. DS)	0.29 (0.095–0.936)	0.038	0.30 (0.09–0.97)	0.045
No of cartridges (vs. SS)	1.62 (1.09–2.40)	0.016	–	–
≤ 2 cartridges (vs. RISS)	2.46 (0.58–8.17)	0.246	–	–
≥ 3 cartridges (vs. RISS)	4.92 (1.43–16.8)	0.011	–	–
Sex (vs. female)	0.64 (0.22–1.88)	0.424	–	–
Age, yr	1.03 (0.99–1.08)	0.113	–	–
BMI, kg/m^2^	1.06 (0.95–1.18)	0.247	–	–
Clinically relevant comorbidities	3.11 (1.13–8.54)	0.028	3.16 (1.13–8.81)	0.028
ASA grade (versus ASA I)	1.58 (0.34–7.31)	0.556	–	–
Smoking	0.66 (0.20–2.18)	0.504	–	–
Distance from the DL, cm	1.12 (0.91–1.37)	0.276	–	–
Neoadjuvant radiotherapy	1.39 (0.52–3.69)	0.505	1.21 (0.44–3.36)	0.703
Robotic assisted (vs. laparoscopy)	0.60 (0.22–1.62)	0.317	–	–
Operative time, min	1.00 (0.99–1.00)	0.984	–	–
Pathological stage (vs. stage 0)	1.64 (0.45–5.94)	0.451	–	–
Operating surgeon (Surgeon 1–5)	–	0.464	–	–

*ASA* American Society of Anesthesiologists; *BMI* body mass index; *DL* dentate line; MD *mean deviation*; *RISS* robotic intracorporeal single-stapled

The statistical analysis was performed using a multivariable binary logistic regression model. The model was statistically significant (χ^2^[3] = 10.51; *P *= 0.015) and explained 11.7% (Nagelkerke’s R^2^) of the variance in 90-day anastomotic leakage, correctly classifying 88% of the cases. The Hosmer–Lemeshow test indicated a good model fit (χ^2^[6] = 4.73; *P *= 0.579)

The number of stapler firings was analyzed both as a continuous and as a categorical variable (≥ 3 vs. < 3 or RISS) in separate models to avoid collinearity with anastomotic type. The alternative model including these variables is presented in Supplementary Table S1

The number of stapler firings was analyzed as both a continuous and a categorical variable (≥ 3 vs. ≤ 2) in separate models to avoid the effects of collinearity. Univariable analysis indicated that the total number of firings (OR 1.62; 95% CI 1.09–2.40; *P *= 0.016) and ≥3 cartridges (versus the use of single staples; OR 4.92; 95% CI 1.43–16.8; *P *= 0.011) were significant predictors of AL. In multivariable models, both the number of continuous stapler firings (OR 1.6; 95% CI 1.06–2.39; *P *= 0.022) (Supplementary Table S1).

## Discussion

Robotic intracorporeal single-stapled anastomosis (RISS) demonstrated significantly lower rates of anastomotic leakage (AL) than did the conventional double-stapled (DS) technique following minimally invasive total mesorectal excision (TME). Moreover, RISS was associated with a reduced need for reintervention, lower postoperative morbidity, and a shorter hospital stay, confirming its favorable technical and clinical profile.

The DS technique is associated with higher AL rates, particularly in ultralow anastomoses, with incidences ranging from 12 to 20%, exceeding those reported with alternative approaches.^26^^,^^27^ These findings reinforce the idea that cross-stapling and the frequent requirement for multiple firings—often unavoidable in a narrow pelvis—compromise perfusion and the integrity of the anastomotic ring, constituting independent risk factors for AL.^26–30^ In addition, the formation of dog ears, inherent to DS, creates tension and ischemic zones along the staple line, a circumstance already associated with an increased risk of failure; technical modifications that eliminated this defect significantly reduced AL in a randomized trial.^31^ Consistently, the Food and Drug Administration (FDA), in its recommendations regarding the use of surgical staplers, has acknowledged the increased risk of leakage in crossed staple lines and advised that cross-stapling be avoided whenever possible.^32^

Transanal transection and single-stapled anastomosis has emerged as an effective alternative. In a *European Journal of Surgical Oncology* study (2021), patients undergoing TTSS had an AL rate of only 2% compared with 17.5% in the DS group and 6% in the TaTME group (*P *= 0.005), as well as a lower reintervention rate (2% vs. 12.6%; *P *= 0.003).^3^ Subsequently, a multicenter study published in *Surgery* (2023) reinforced these findings: TTSS achieved an AL rate of 6.48%, which was significantly lower than the 15.28% observed with DS (*P *= 0.002) and was also associated with a lower severity of anastomotic complications.^1^ These results, which are consistent across different contexts, support the technical principle that a single stapling and a complete circular ring result in safer anastomoses.

Robotic intracorporeal single-stapled anastomosis is an evolution of the single-stapled concept, integrating single-staple anastomosis into the robotic environment and optimizing the execution of hand-sewn purse-string anastomosis. High-resolution three-dimensional pelvic visualization, alternating 30° optical focus, articulated instruments with multiple degrees of freedom, and enhanced ergonomics are essential attributes for surgical precision at this stage, which is unfeasible for intracorporeal laparoscopy. The global dissemination of robotic platforms supports the adoption of this technique in multiple specialized centers, favoring reproducibility and standardization.

In contrast, although TTSS achieves excellent outcomes, it requires advanced proficiency in the perineal approach and TaTME skills, which may limit its scalability. Recent studies, including a multicenter randomized trial published in *JAMA* (2025), demonstrated that robotic TME offers advantages over laparoscopy for mid- and low-rectal cancer, including reduced locoregional recurrence (1.6% vs. 4%; adjusted hazard ratio, 0.39; *P *= 0.03), improved disease-free survival (87.2% vs. 83.4%; *P *= 0.04), and superior preservation of urinary and sexual function.^14^ These findings support the hypothesis that an intracorporeal robotic approach, such as that involved in RISS, can seamlessly follow TME dissection to allow precise transection and anastomotic construction in a single operative step without the need for redocking, instrument withdrawal, or an additional perineal approach, thus achieving desirable safety, precision, and reproducibility.

In our initial experience,^15^ we standardized a stepwise protocol for isolation and presentation of the tumor-free rectal stump, facilitating direct and symmetric intracorporeal transection, followed by robotic-assisted anastomosis. This standardization proved critical, because no intracorporeal robotic systematizations were previously described, thereby allowing a reproducible learning curve.

Prior studies have demonstrated that TTSS enables more precise distal margins by performing transection under direct visualization, minimizing the risk of oncologic compromise in ultralow tumors. In addition, single stapling ensures complete 360° circumferential closure, eliminating dog ears and improving staple-line safety. These benefits are reproduced in RISS, which is now enhanced by robotic ergonomics in the pelvic environment. In our series, compared with those in the DS group, the tumors in the RISS group were significantly closer to the anal verge (5.08 cm vs. 6.27 cm; *P *< 0.001). This outcome may reflect selection bias, because lower tumors were preferentially managed with robotic visual transection, whereas patients who required hand-sewn coloanal anastomoses—a prespecified exclusion criterion—were not included.^33^ Nevertheless, RISS appears particularly advantageous for low extraperitoneal tumors, where technical precision and rectal stump presentation through a plug (gauze in a surgical glove or the circular stapler itself) allow an intact, circular, and symmetric transection, preserving an ideal stump for anastomotic construction.

Rectal opening is an established component of *TaTME* and *TTSS* procedures, both of which have been validated regarding local oncologic safety and recurrence^3^^,^^11–13^ The RISS technique adheres to these same oncologic principles, with distal transection performed under direct visualization, ensuring a more accurate distal margin than that obtained with the conventional DS technique, in which the linear stapler is applied blindly and obliquely in narrow pelvises.

In our results, no local recurrence has been identified to date. The lower postoperative morbidity observed with RISS may also be associated with improved long-term oncologic prognosis, as postoperative complications have been consistently linked to adverse survival outcomes.^34–36^ Ongoing surveillance will provide additional long-term data on oncologic outcomes.

Another relevant finding was the direct association between the number of stapler firings and AL risk. The mean number was 2.42 ± 0.65, and multiple firings represented an independent risk factor, whereas RISS was protective. A continuous analysis revealed that each additional firing progressively increased the risk of AL (OR 1.62; 95% CI 1.09–2.40; *P *= 0.016). When categorized, the use of ≥ 3 firings emerged as an independent risk factor (OR 4.98; 95% CI 1.38–18.0*; P *= 0.014), whereas ≤ 2 firings did not reach significance, despite a trend toward increased risk (OR 2.19; 95% CI 0.57–8.34; *p *= 0.14).

In our analysis, compared with RISS, DS was associated with higher overall morbidity (52.5% vs. 33.3%; *P *= 0.014) and a greater need for surgical reintervention (10.4% vs. 1.4%; *P *= 0.025). These findings are consistent with those of the multicenter study by Gamboa et al., published in *Annals of Surgical Oncology*, which included more than 1,500 patients who underwent rectal resection with DS and reported complications in 46% of the cohort, of which 63% were minor and 32% major, further showing that major complications negatively impact both recurrence-free and overall survival.^34^ Similarly, in a nationwide analysis of nearly 17,000 patients in the Netherlands, Warps et al. demonstrated that the occurrence of any postoperative complication was associated with significantly reduced 5-year survival, with a particularly pronounced effect for nonsurgical and combined complications.^35^ More recently, Aoyama et al.,^36^ in a study published in *Annals of Oncology*, reported that complications, such as AL, pneumonia, and surgical site infection significantly compromise both clinical and oncologic outcomes.^36^^,^^37^ Collectively, this evidence suggests that the reduced morbidity and fewer reinterventions observed with RISS may translate into potential long-term oncologic advantages.

At our institution, early endoscopic evaluation with flexible sigmoidoscopy is routinely performed between 2 and 3 weeks postoperatively, which may have contributed to the increased detection of grade A leaks according to the International Study Group of Rectal Cancer (ISREC) classification.^10^ Although such leaks do not alter management, they explain the predominance of early (< 30 days) complications in our series. All leaks in the RISS group occurred within 30 days, whereas the DS group had a 14.6% rate within the same interval. These findings underscore that follow-up protocols influence the detection of complications, particularly subclinical complications, and should be considered when different series are compared.

Importantly, our analysis followed the most recent international recommendations. The International Consensus on Reporting Anastomotic Leaks After Colorectal Cancer Surgery (CoReAL), published in 2025, established 33 evidence-based statements and 43 elements for standardized reporting of anastomotic leaks.^16^ Another relevant point concerns the broader applicability of the technique. In a report published in *Colorectal Disease* (2024), we described the use of robotic hand-sewn purse-string anastomosis to manage colorectal anastomotic stricture following prior DS, achieving technical success without complications. This finding reinforces the versatility of the approach and demonstrates its utility in complex reconstructive scenarios where DS would be unsuitable for adequate reanastomosis.^38^

Among the limitations of this study, some patients in the DS cohort underwent surgery laparoscopically (44.8%). However, when comparing robotic-assisted DS versus laparoscopic DS, no significant differences were observed (OR 0.6; 95% CI 0.22–1.62; *P *= 0.31). Similarly, a meta-analysis by Asmat et al.,^39^ based on randomized controlled trials and including 2,525 patients, revealed no differences between robotic and laparoscopic TME regarding AL, severe complications (Clavien–Dindo III–V), or mortality, reinforcing the comparable safety of both approaches.

Additionally, the relatively small sample size and the low number of anastomotic leak events inherently limit the statistical power and robustness of the multivariable analysis. In accordance with standard methodological guidance for logistic regression with few outcome events, we restricted each model to a maximum of three covariates and constructed alternative models to avoid overparameterization. Variables with established clinical relevance were prioritized, and potentially collinear factors, such as the anastomotic type and the number of stapler firings, were not included simultaneously. Although this approach minimizes overfitting, the results should be interpreted with caution and regarded as hypothesis-generating rather than confirmatory.

Compared with reconstructions performed exclusively perineally, RISS has the potential to facilitate the creation of higher anastomoses because of the wide endopelvic visual field and adequate rectal stump exposure. This suggests greater technical versatility, enabling safe margins and facilitating rectal transection in higher reconstructions. Additional studies, preferably randomized trials directly comparing RISS and TTSS, are warranted to confirm this hypothesis, including subgroup analyses in specific populations, such as morbidly obese patients. In our series, the median distance from the anastomosis to the dentate line was 2.37 cm in patients with mid-rectal tumors.

Although initial operative times for RISS were longer, reflecting the learning process inherent to a new technique, morbidity did not increase. With accumulated experience, a progressive reduction in operative time was observed, as also demonstrated in the CUSUM analysis (Supplementary Fig. 1), reaching a stable plateau consistent with procedural standardization. Six surgeons were involved in both techniques, with no significant intersurgeon differences (*P *= 0.464), supporting the reproducibility of the RISS approach during its adoption phase. Furthermore, the combination of lower complication rates, fewer reinterventions, shorter hospitalization, and the avoidance of linear staplers and additional cartridges suggests that RISS may offer a cost-efficient alternative to DS.

As with any retrospective comparison, potential selection bias cannot be completely excluded. However, baseline and operative variables were largely comparable between groups, and multivariable analysis confirmed the absence of major imbalances in known risk factors for anastomotic leakage. Although this design provides valuable real-world evidence, definitive conclusions will require prospective and ideally randomized trials to validate the superiority of RISS over conventional DS and to further assess its long-term oncologic impact.

In summary, compared with the conventional DS technique, RISS demonstrated superior safety and efficacy, with lower rates of anastomotic leakage and associated complications. This method combines the proven benefits of single-stapling techniques, as previously demonstrated in TTSS, with the ergonomic and precision advantages of robotic platforms, enabling the retention of safe distal margins under direct visualization. Our findings also confirm that multiple stapler firings remain an independent risk factor for AL, reinforcing the recommendation to replace ≥ 3 firings with hand-sewn purse-string suturing via RISS. Prospective multicenter trials, including direct comparisons with TTSS and cost-effectiveness analyses, will be crucial for consolidating the role of RISS and expanding its clinical applicability.

## Supplementary Information

Below is the link to the electronic supplementary material.

Supplementary file1 (MP4 333069 KB)

Supplementary file2 (DOCX 16 KB)

Supplementary Figure 1. Cumulative sum (CUSUM) learning curve of operative time for robotic anterior resection using the Robotic Intracorporeal Single-Stapled (RISS) technique. The curve illustrates an initial learning phase characterized by longer operative times, followed by a progressive improvement phase with decreasing durations, and a plateau phase consistent with attainment of technical proficiency (TIFF 66 KB)
