# Supplementary material for: Robotic Intracorporeal Single-Stapled Anastomosis (RISS) is Associated with Lower Anastomotic Leakage Rates than the Double-Stapled Technique After Minimally Invasive Total Mesorectal Excision for Rectal Cancer
Source: Ann Surg Oncol. 2025 Nov 20;33(3):1935–45. doi: 10.1245/s10434-025-18742-3 (PMC12901133; doi:10.1245/s10434-025-18742-3)
Supplement: Supplementary file 2 — Supplementary file2 (DOCX 16 KB) [file 10434_2025_18742_MOESM2_ESM.docx]

**Supplementary Table S1. Alternative Multivariable Model Including Both the Number of Stapler Firings and Anastomotic Type**

| Variable | Univariable analysis | | Multivariable analysis | |
| --- | --- | --- | --- | --- |
|  | OR/MD (95% CI) | P value | OR (95% CI) | P value |
| No of cartridges (versus SS) | 1.62 (1.09–2.40) | 0.016 | 1.60 (1.06–2.39) | 0.022 |
| < 2 cartridges (versus RISS) | 2.46 (0.58–8.17) | 0.246 | – | – |
| > 3 cartridges (versus RISS) | 4.92 (1.43–16.8) | 0.011 | – | – |
| Sex (versus female) | 0.64 (0.22–1.88) | 0.424 | – | – |
| Age, y | 1.03 (0.99–1.08) | 0.113 | – | – |
| BMI, kg/m^2^ | 1.06 (0.95–1.18) | 0.247 | – | – |
| Clinically relevant comorbidities | 3.11 (1.13–8.54) | 0.028 | 3.11 (1.11–8.69) | 0.031 |
| ASA grade (versus ASA I) | 1.58 (0.34–7.31) | 0.556 | – | – |
| Smoking | 0.66 (0.20–2.18) | 0.504 | – | – |
| Distance from the DL, cm | 1.12 (0.91–1.37) | 0.276 | – | – |
| Neoadjuvant radiotherapy | 1.39 (0.52–3.69) | 0.505 | 0.90 (0.32–2.54) | 0.852 |
| Robotic assisted (versus laparoscopy) | 0.60 (0.22–1.62) | 0.317 | – | – |
| Operative time, min | 1.00 (0.99–1.00) | 0.984 | – | – |
| Pathological stage (versus stage 0) | 1.64 (0.45–5.94) | 0.451 | – | – |
| Operating surgeon (Surgeon 1–5) | – | 0.464 | – | – |

Abbreviations: ASA: American society of anesthesiologists; BMI: body mass index; DL: dentate line; MD: mean deviation; RISS: robotic intracorporeal single-stapled.

Notes:

The statistical analysis was performed using a multivariable binary logistic regression model. The model was statistically significant (χ²[3] = 11,56; p = 0.009) and explained 12.9% (Nagelkerke’s R²) of the variance in 90-day anastomotic leakage, correctly classifying 88% of the cases. The Hosmer–Lemeshow test indicated a good model fit (χ²[6] = 6.17; p = 0.63).
