# Supplementary figures and images for: Robotic Intracorporeal Single-Stapled Anastomosis (RISS) is Associated with Lower Anastomotic Leakage Rates than the Double-Stapled Technique After Minimally Invasive Total Mesorectal Excision for Rectal Cancer
Source: Ann Surg Oncol. 2025 Nov 20;33(3):1935–45. doi: 10.1245/s10434-025-18742-3 (PMC12901133; doi:10.1245/s10434-025-18742-3)

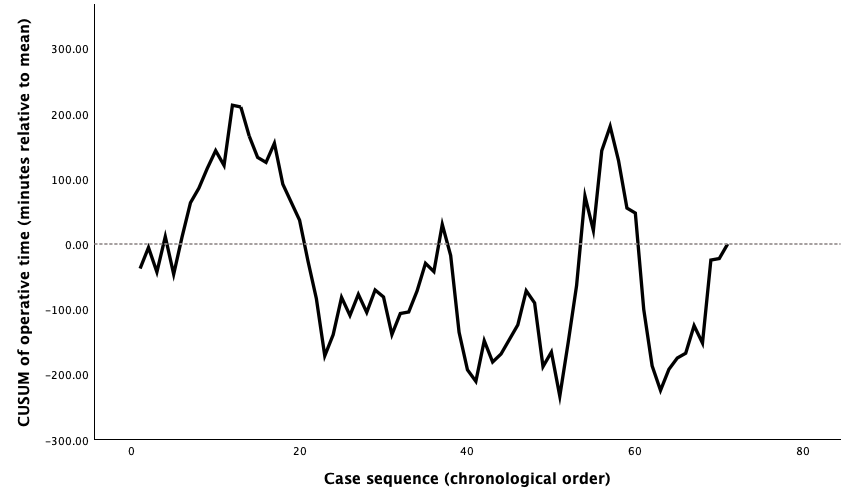

Supplement: Supplementary file 3 — Supplementary Figure 1. Cumulative sum (CUSUM) learning curve of operative time for robotic anterior resection using the Robotic Intracorporeal Single-Stapled (RISS) technique. The curve illustrates an initial learning phase characterized by longer operative times, followed by a progressive improvement phase with decreasing durations, and a plateau phase consistent with attainment of technical proficiency (TIFF 66 KB) [file 10434_2025_18742_MOESM3_ESM.tiff]
